# Rare complication of internal jugular vein catheter ectopically placed into the dural sinus: a case report

**DOI:** 10.3389/fped.2026.1796431

**Published:** 2026-04-20

**Authors:** Lu Wang, Yan Jiang

**Affiliations:** Pediatric Emergency Center, Department 1, Gansu Maternal and Child Health Hospital, Lanzhou, Gansu, China

**Keywords:** catheter tip malposition, chemical meningitis, dural sinus, imaging examination, internal jugular vein catheterization, ultrasound

## Abstract

**Background:**

Central venous catheterization is a procedure involving the percutaneous insertion of a catheter into a central vein, such as the internal jugular, subclavian, or femoral vein. It is frequently used for monitoring central venous pressure (CVP), assessing central venous oxygen saturation (ScvO_2_), performing blood purification, providing nutritional support, and administering specialized medications. Among available sites, the internal jugular vein is commonly preferred. Catheter malposition is a recognized complication. Malpositioned catheters typically occur in larger veins, including the internal jugular, axillary, subclavian, and innominate veins. Rarely, ectopic catheter placements occur within smaller vessels associated with the venous trunk, venous sinuses, lymphatic vessels, or, exceptionally, within the arterial system. This article reports a rare case of an internal jugular vein catheter malpositioned intracranially, resulting in infiltration of intravenous nutrient fluid into the cerebrospinal fluid (CSF). The report aims to provide clinical guidance and raise awareness among medical personnel regarding this uncommon complication.

**Case summary:**

A 6-month-old boy was admitted to the pediatric surgery department for colostomy reversal. Before the operation, internal jugular vein catheterization was successfully performed under ultrasound guidance. However, on the second day after catheter placement, no blood return was obtained from the central venous catheter (CVC). Cervical vascular ultrasound initially suggested normal catheter positioning within the internal jugular vein. On the fourth day after catheterization, the patient developed poor oxygen saturation and seizures. Comprehensive evaluations, including CSF analysis, cervical vascular ultrasound, and radiographs of the skull and chest, strongly suggested that the CVC had inadvertently entered the intracranial dural sinus, causing intravenous nutrient fluid infiltration into the CSF. The patient was managed aggressively with endotracheal intubation, mechanical ventilation, antibiotic therapy, and measures to reduce intracranial pressure. Following treatment, he recovered fully and was discharged from the hospital.

**Conclusion:**

Precise technique and accurate catheter positioning are essential for successful internal jugular vein catheterization. Although ultrasound guidance improves catheterization success rates, it has significant limitations in preventing distal catheter-tip malposition. Therefore, imaging examinations remain critical to confirm catheter placement and trajectory, helping to prevent potential complications.

## Introduction

Central venous catheters (CVCs) are widely used in clinical practice for monitoring CVP, ScvO_2_, performing blood purification, providing nutritional support, and administering specialized therapies. Among the available access routes, the internal jugular vein is commonly selected for cannulation. Catheter tip malposition is a common complication of CVC insertion. Malpositioned catheters are most often located in large central veins, including the internal jugular, axillary, subclavian, and innominate veins (1). In contrast, rare malpositions may occur in smaller vessels connected to the venous trunk, venous sinuses, lymphatic vessels, or, in rare cases, the arterial system. Globally, approximately 27 million CVCs are placed annually (2). Despite their widespread use, complications remain frequent, including catheterization failure, inadvertent arterial puncture, vascular injury, pneumothorax, catheter malfunction, catheter-related bloodstream infections, and deep vein thrombosis (3). This article reports a rare case of CVC malposition to assist clinicians in recognizing and managing uncommon ectopic placements.

## Case presentation

The patient was a 6-month-old male admitted for colostomy closure. He was diagnosed with congenital anal atresia with a rectourethral fistula at birth. A stage I transverse colon double-lumen colostomy was performed 2 days after birth. At 3 months of age, laparoscopic repair of the rectourethral fistula and perineal anoplasty were performed. Internal jugular vein catheterization had been performed during both procedures without complications. The patient was born at term by cesarean section (G2P2), with a birth weight of 2.18 kg. His parents and elder sister were healthy, and no family history of genetic disease was reported.

### Physical examination

Vital signs were stable. The patient's height was 61.4 cm, and weight was 6.9 kg. No abnormalities were observed in the neck. Bilateral colostomy stomas were located on the inner side of the left upper abdomen along the midclavicular line. The colonic mucosa at the stoma sites appeared erythematous, with a small amount of adherent stool. The anal mucosa was also erythematous, with no evidence of retraction. No other abnormalities were detected.

### Auxiliary examination

Blood tests performed after admission were within normal ranges. Chest and abdominal radiographs, as well as colonography, showed no abnormalities. Brain MRI with MR venography revealed no significant findings.

### Diagnostic process

Prior to colostomy closure, ultrasound-guided internal jugular vein catheterization was performed by the anesthesiologist. A 22G single-lumen catheter, 8 cm in length, was inserted. The procedure was initially successful, with free blood return observed. No imaging was performed to confirm catheter tip position. Postoperatively, cefotaxime was administered for infection prophylaxis, and the patient was maintained nil per os and received total parenteral nutrition (Table 1).

**Table 1:** Timeline of the diagnosis and treatment process.

Time node	Event description
2025-5-30	Ultrasound-guided internal jugular vein catheterization was performed using a 22G single-lumen catheter (8 cm in length).
2025-6-1	No blood return was obtained from the CVC. Vascular ultrasound showed normal positioning of the proximal and middle segments.
2025-6-3	The patient developed hypoxia and convulsions. Examination revealed that the CVC had entered the intracranial cavity. Infusion through the ectopic CVC was immediately discontinued, and treatment was initiated, including infection control, reduction of intracranial pressure, and repeated lumbar punctures for CSF drainage.
2025-6-17	The patient achieved clinical recovery. CSF returned to normal, and no abnormalities were detected on head MRI. The patient was discharged in stable condition.
2025-7-20	At 1-month follow-up after discharge, no complications were observed.
2025-9-20	At 3-month follow-up, normal growth and development were observed.

During recovery, intermittent fever and cough were noted. On postoperative day 2, no blood return was obtained during aspiration. Vascular ultrasound showed normal positioning of the proximal and middle catheter segments, and the catheter was retained for continued use. On postoperative day 4, cyanosis of the lips developed, and oxygen saturation decreased to approximately 56%. Oxygen via nasal cannula was ineffective. Emergency interventions included bag-assisted ventilation, sputum suction, and tracheal intubation. During this period, convulsions occurred and were controlled with intravenous midazolam. The patient was transferred to the Pediatric Intensive Care Unit (PICU) for further management.

After PICU admission, lumbar puncture was performed, yielding milky and turbid CSF. CSF samples were sent for routine, biochemical, and culture analyses. Routine examination showed a white blood cell count of 28 × 10^6^/L, with 82% polymorphonuclear cells. Biochemical analysis revealed glucose of 60.64 mmol/L, chloride of 86 mmol/L, and total protein of 0.38 g/L. CSF culture was negative. The markedly elevated CSF glucose suggested leakage of hypertonic nutritional solution into the CSF. Laboratory tests showed CRP of 6.42 mg/L and PCT of 1.38 ng/mL, while blood cultures were negative.

**Figure 1:**
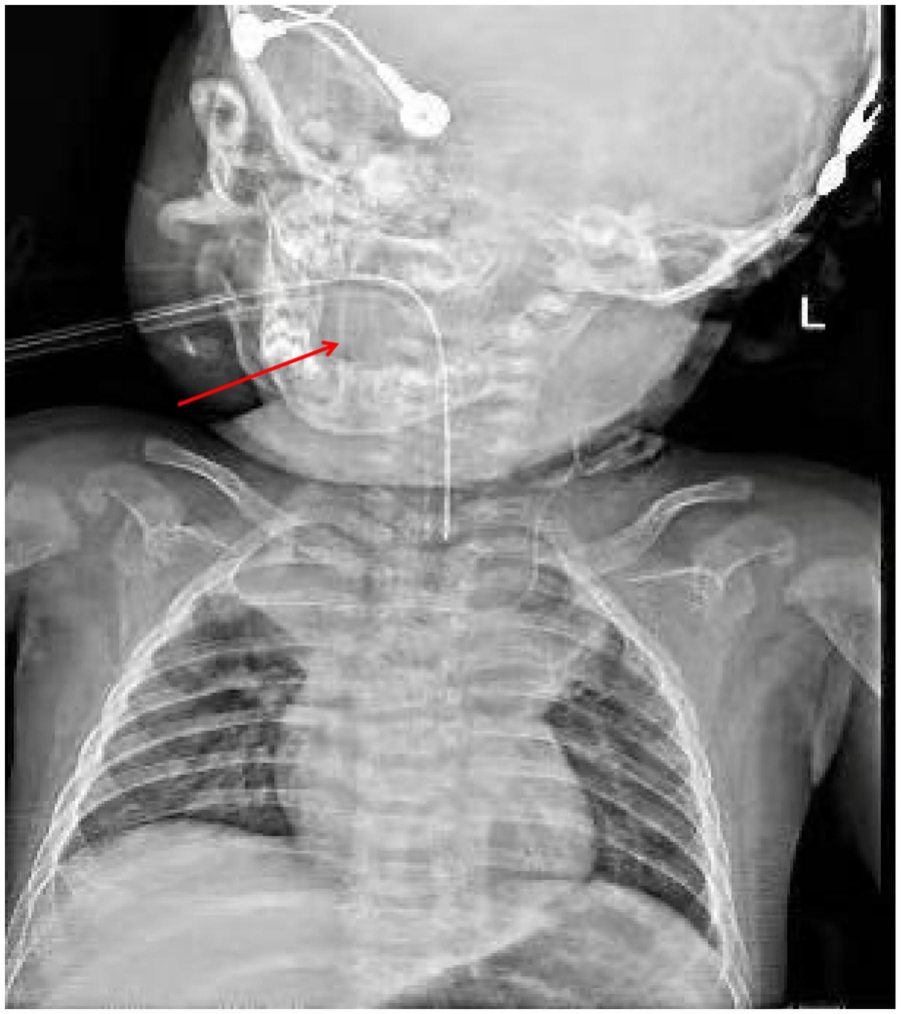
Catheter tip positioned within the intracranial cavity (indicated by the arrow).

Chest x-ray (CXR) showed bilateral pulmonary exudation with partial consolidation, and no catheter was visualized. Vascular ultrasound revealed that the catheter did not extend distally within the right internal jugular vein. Cranial ultrasound suggested bilateral hydrocephalus. Chest and cranial x-rays confirmed upward malposition of the catheter into the intracranial cavity (Figure 1).

### Treatment

The ectopic CVC infusion was immediately discontinued. A new catheter was inserted into the left internal jugular vein. Treatment included ceftriaxone sodium for infection control, mannitol to reduce intracranial pressure, and repeated lumbar punctures for CSF drainage. The patient recovered clinically and was discharged without complications.

### Follow-up

At 1-month follow-up, no complications were observed. At 3 months, the child showed normal growth and development.

## Discussion

Globally, approximately 27 million CVCs are placed annually for various clinical purposes (2). In recent years, the application of bedside ultrasound has markedly improved the success rate of catheter placement and reduced the incidence of mechanical complications (4, 5). However, complications can still arise and may pose life-threatening risks. Mechanical complications include catheterization failure, inadvertent arterial puncture, catheter tip malposition, bleeding, symptomatic arrhythmia, pneumothorax, and persistent nerve damage. Early detection and timely management of catheter tip malposition usually prevent adverse events. Conversely, delayed recognition can lead to severe, unpredictable complications during fluid infusion, as demonstrated in the present case.

Central venous catheterization is frequently necessary for critically ill patients, but it carries a relatively high complication rate. Pediatric patients often present additional challenges during catheter placement, especially those with congenital anomalies. An increased incidence of anatomical variations, including anomalies of central veins, is observed in this population. Therefore, an evaluation of the anatomical condition, depth, and any potential variations of veins should be performed before catheterization. The Rapid Central Venous Assessment (RaCeVA) protocol provides a structured ultrasound approach to evaluate veins in the neck and supraclavicular/subclavian areas prior to central intravenous catheter placement (CICC). This protocol is beneficial for teaching various ultrasound-guided venous access techniques and ensures systematic consideration of all potential veins, enabling operators to select the optimal access site (6). Uzumcugil et al. (7) conducted a prospective study involving children aged 0–2 years, demonstrating substantial variability in the position of the right internal jugular vein. Their findings highlight the importance of applying the RaCeVA protocol before catheter insertion. Although the child in this case previously underwent two uncomplicated internal jugular vein catheterizations, the patient was a young infant with congenital anal atresia. Therefore, performing a rapid central vein assessment prior to the procedure could have reduced the risk of complications.

The optimal position for the catheter tip has long been debated. The generally accepted ideal location for CVC placement is at the distal third of the superior vena cava, near the cavoatrial junction. This site is thought to minimize complications such as vascular perforation, local thrombosis, catheter dysfunction, and retrograde intracranial infusion (8, 9). Therefore, accurate catheter tip positioning is essential for safe and effective CVC use. Methods to confirm tip placement include intracavitary electrocardiography, CXR, and echocardiography. Ultrasound-guided deep vein catheterization allows clinicians to identify the target vessel, detect anatomical variations and thrombosis at the insertion site, and improve puncture success rates (3). However, ultrasound has significant limitations in identifying distal catheter tip malposition. CXR remains the most common and well-recognized method to verify CVC tip placement, despite its inherent limitations, including radiation exposure, delayed results, and inability to make real-time adjustments. Some studies suggest that routine CXR might be unnecessary for tip confirmation given the low complication rate associated with ultrasound-guided procedures (10). However, research by Camporesi et al. (11), assessing pediatric CVC placements worldwide, emphasized adopting standardized methods for catheter tip verification, despite varying resources and clinical practices. Although ongoing debates continue regarding routine CXR after ultrasound-guided catheterization, chest radiography remains the gold standard. CXR should be utilized whenever difficulties arise during catheter placement, operator uncertainty exists, or ultrasound results are ambiguous. In this case, initial catheter insertion was successful, but blood return subsequently ceased. At that point, CXR should have been performed promptly to verify catheter position.

Retrospective studies reported a catheter dislocation rate of approximately 1.5% in pediatric patients (12). The incidence of dural sinus thrombosis is even lower, at approximately 1.1 cases per 100,000 annually (13). In rare cases, internal jugular vein catheterization can result in the catheter tip folding backward and entering intracranial dural sinuses, such as the sigmoid or transverse sinus. Anatomically, the sigmoid sinus lies between the dural layers within the temporal bone's sigmoid sulcus, crossing anteriorly over the jugular process, terminating at the jugular foramen and internal jugular bulb. Its intracranial continuation, the transverse sinus, runs along the posterior cerebellar margin, following the transverse occipital sulcus, and curves downward into the sigmoid sulcus at the attachment of the tentorium cerebelli to the petrous temporal bone. Such intracranial catheter malpositions are extremely rare, with only a few reported cases. Ibrahim (14) described a case in which an internal jugular catheter inadvertently entered the sigmoid sinus; fortunately, timely detection prevented complications. In the present case, the catheter tip likely entered the dural sinus, causing leakage of intravenous nutrient fluid into the CSF and resulting in chemical meningitis. Prompt recognition and management enabled full recovery. The most likely mechanism was initial correct catheter placement in the internal jugular vein, followed by upward folding and reversal of the catheter tip. Under negative thoracic pressure, the reversed catheter advanced intracranially. Considering potential risks, only head and CXRs were conducted to confirm intracranial catheter location, after which the catheter was removed. CT or MRI was not performed at the time for precise localization, but imaging combined with clinical presentation strongly suggested dural sinus entry. Chemical meningitis can occur via two primary mechanisms: (1) hypersensitivity reactions and (2) direct chemical irritation of the meninges, typically involving direct injection of substances into the CSF (15). Chemical meningitis usually presents with negative CRP, PCT, and bacterial cultures, significantly elevated CSF protein, and relatively normal glucose. In this case, the CSF appeared milky-white and turbid, had slightly elevated cell counts, no substantial elevation of CRP or PCT, and negative bacterial cultures. These findings supported a diagnosis of chemical meningitis caused by direct chemical irritation from the intravenous nutrient solution. The infused nutrient solution had a glucose concentration of 10%, significantly higher than normal CSF glucose levels. Once this solution entered the subarachnoid space, it resulted in markedly elevated CSF glucose levels. Fortunately, immediate discontinuation of infusion, appropriate anti-infective therapy, and intracranial pressure reduction measures enabled complete recovery without neurological sequelae.

## Conclusion

Although uncommon, ectopic CVC placement can result in severe and potentially life-threatening complications (16). Therefore, catheter insertion should be performed by experienced clinicians, ideally under ultrasound guidance. Post-procedural imaging, such as CXR, is recommended to verify catheter tip position. Any sign of catheter dysfunction during use should prompt immediate radiographic assessment to confirm tip location and prevent serious complications.

## Data Availability

The original contributions presented in the study are included in the article/Supplementary Material, further inquiries can be directed to the corresponding author/s.
